# Dietary supplementation with rice bran fermented with *Lentinus edodes* increases interferon-γ activity without causing adverse effects: a randomized, double-blind, placebo-controlled, parallel-group study

**DOI:** 10.1186/1475-2891-13-35

**Published:** 2014-04-22

**Authors:** Ji-young Choi, Doo-Jin Paik, Dae Young Kwon, Yongsoon Park

**Affiliations:** 1 Department of Food and Nutrition, Hanyang University, Seoul, Korea; 2 Department of Anatomy and Cell Biology, College of Medicine, Hanyang University, Seoul, Korea; 3 Emerging Innovative Technology Research Division, Korean Food Research Institutes, Sungnam, Korea

**Keywords:** Cytokines, Fermented rice bran, Immune response, Natural killer cell, Randomized clinical study

## Abstract

**Background:**

The purpose of this study was to investigate the hypothesis that dietary supplementation with rice bran fermented with *Lentinus edodes* (rice bran exo-biopolymer, RBEP), a substance known to contain arabinoxylan, enhances natural killer (NK) cell activity and modulates cytokine production in healthy adults.

**Methods:**

This study was designed in a randomized, double-blind, placebo-controlled, and parallel-group format. Eighty healthy participants with white blood cell counts of 4,000-8,000 cells/μL were randomly assigned to take six capsules per day of either 3 g RBEP or 3 g placebo for 8 weeks. Three participants in the placebo group were excluded after initiation of the protocol; no severe adverse effects from RBEP supplementation were reported. NK cell activity of peripheral blood mononuclear cells was measured using nonradioactive cytotoxicity assay kits and serum cytokine concentrations included interferon (IFN)-γ, tumor necrosis factor (TNF)-α, interleukin (IL)-2, IL-4, IL-10, and IL-12 were measured by Bio-Plex cytokine assay kit. This study was registered with the Clinical Research Information Service (KCT0000536).

**Results:**

Supplementation of RBEP significantly increased IFN-γ production compared with the placebo group (*P* = 0.012). However, RBEP supplementation did not affect either NK cell activity or cytokine levels, including IL-2, IL-4, IL-10, IL-12, and TNF-α, compared with the placebo group.

**Conclusions:**

The data obtained in this study indicate that RBEP supplementation increases IFN-γ secretion without causing significant adverse effects, and thus may be beneficial to healthy individuals. This new rice bran-derived product may therefore be potentially useful to include in the formulation of solid and liquid foods designed for treatment and prevention of pathological states associated with defective immune responses.

## Background

Rice bran, a major byproduct of rice processing, is currently an underutilized resource even though it is an excellent source of valuable bioactive compounds such as dietary fiber, vitamins, and antioxidants [1]. Among the dietary fibers contained in rice bran, arabinoxylan, pectin, and β-glucan have been suggested to be functional polysaccharides with biological response modifier (BRM) properties [2]. For instance, polysaccharides from rice bran have been shown to activate immunological cells which comprise part of the body’s front line of defense against cancer [3,4]. However, the functionality of immune-enhancing BRMs in rice bran is limited, since BRMs are located in the cell wall where they are complexed with hemicellulose [5].

Enzymatic processes have been developed that separate biologically active polysaccharides from the rest of the rice bran, so that these polysaccharides can be exploited as new BRMs [6,7]. Rice bran fermented with *Lentinus edodes* (rice bran exo-biopolymer, RBEP) is a food supplement that is obtained by reacting rice bran with Shiitake mushroom-derived carbohydrates [8]. Furthermore, RBEP has been shown to suppress the growth of melanoma cancer cells by enhancing natural killer (NK) cell activity in mice [2] and by activating macrophage cells both *in vivo* and *in vitro*[5,8]. Different products derived from rice bran and mushroom cocultures have also been reported to increase NK cell activity in patients with multiple myeloma [9], cancerous mice [10], and lymphocytes [11,12]. In particular, nutritional supplementation with arabinoxylan, a rice bran-derived polysaccharide, was shown to increase NK cell activity in patients with various cancers [13].

NK cells play a crucial role in host anti-cancer defense, and thus are tightly regulated by multiple mechanisms. Activation of NK cells to kill target cells can be both directly and indirectly modulated by cytokines, including interferon (IFN), interleukins (ILs), and tumor necrosis factor (TNF) [14]. Previous studies with products derived from rice bran and mushroom cocultures also demonstrated increased concentrations of IFN-γ and TNF-α in patients with multiple myeloma [9], in addition to Ehrlich carcinoma-bearing mice [10]. However, no clinical studies of healthy individuals have yet been performed to investigate the effects of RBEP supplementation on NK cell activity and cytokine concentrations. Therefore, this randomized, double-blind, placebo-controlled, parallel-group study was designed to test the hypothesis that RBEP supplementation enhances NK cell activation and modulates cytokine production in healthy Korean individuals.

## Methods

### Study materials

RBEP and placebo were prepared by Erom Corporation (Sungnam, Korea) according to the method reported by Yu et al. [8]. Briefly, *Lentinus edodes* was cocultured in its appropriate growth medium with rice bran until significant mycelia growth was observed. Next, *Lentinus edodes* mycelia and insoluble rice bran components were removed by centrifugation. Using alkali extraction and ethanol precipitation, polysaccharides were collected from the resultant supernatants and then lyophilized. Uronic acid was used as a marker compound for RBEP. Table 1 shows the chemical composition of RBEP. The placebo contained 430.69 mg cornstarch, 69.29 mg food coloring, and 2.51 mg stearic acid. Six capsules of either 3 g RBEP or placebo were provided daily.

**Table 1:** **Chemical composition of the fermentation product of rice bran and *Lentinus edodes***

**Constituent**	**Composition (%)**
Carbohydrates	63.2
Protein	17.6
Fat	4.1
Moisture	4.4
Ash	10.7
Uronic acid	5.2
Carbohydrate components	Composition (%)
Glucose	11.71
Xylose	22.25
Galactose	Trace
Arabinose	Trace

### Study design

This study was an 8-week, randomized, double-blind, placebo-controlled, and parallel-group clinical study. All work described here was approved by the Institutional Review Board of Hanyang University Hospital (HYUH 2012-05-008). The present study was performed in accordance with the Declaration of Helsinki, and written informed consent was obtained from all participants. This study was registered with the Clinical Research Information Service (KCT0000536).

In total, each patient underwent 1 screening and 3 visits in this study. At the initial screening, participants were interviewed to collect sociodemographics data and medical history was obtained through an interview, and his/her white blood cell (WBC) count was measured. Height was measured without shoes using a stadiometer, and weight was measured while participants were wearing light clothes without shoes using an Inbody 720 (Biospace Corporation, Seoul, Korea). Visits occurred at the following times after the initial screening: visit 1 (week 0), 7–10 days postscreening; visit 2, 4 weeks postscreening; and visit 3, 8 weeks postscreening. At all visits, outcome measurements were taken and safety assessments were performed. Fasting blood and urine samples were collected and stored at -20°C until analysis. At weeks 4 and 8, compliance was monitored by counting the number of remaining capsules.

Participants were asked not to take any health products containing fermented rice bran, and not to change their usual lifestyle and diet during the study. Three-day dietary records were used to monitor changes in diets at weeks 0, 4, and 8; these records were analyzed with CAN-pro 4.0 (Computer Aided Analysis Program 4.0 for professionals, Korean Society of Nutrition, Seoul, Korea).

### Participants

Participants were recruited through poster and newspaper advertisements during September 2012. One hundred and sixty-three volunteers were screened, and 80 participants were eligible to participate in this study. Participants were included if they were 25–70 years old, not pregnant or lactating, and had a WBC count between 4,000-8,000 cells/μL. Participants were excluded if they had any infectious disease; a chronic disease such as cardiovascular disease, diabetes mellitus, kidney disease, thyroid disease, or a psychiatric disorder; or had taken any medications or supplements regularly during the previous 3 months. Other exclusion criteria included having a creatinine level ≥ 2 times the normal upper limit, and having an aspartate aminotransferase (AST) or alanine aminotransferase (ALT) level ≥ 3 times the normal upper limit.

### Outcome measurements

NK cell activity and cytokine concentrations included IFN-γ, TNF-α, IL-2, IL-4, IL-10, and IL-12 were measured at weeks 0, 4, and 8. To measure NK cell activity, peripheral blood mononuclear cells (PBMCs) were prepared by density gradient separation. Pelleted cells were resuspended in phosphate-buffered saline, and their viabilities were determined using trypan blue solution. To measure NK cell activity, nonradioactive cytotoxicity assay kits (Promega Inc., Madison, WI, USA) were used. Effector cells (PBMCs) were seeded in 96-well plates, with K562 cells (Korean Cell Line Bank, Seoul, Korea) used as target cells. The ratio of effector:target cells was 10:1, and each assay was performed in triplicate. Assays were performed according to the manufacturer’s instructions. Briefly, plates were incubated at 37°C with 5% CO_2_ for 4 hours. Absorbances at 490 nm were then read with an iMark TM microplate reader (Bio-Rad Laboratories, Inc., Hercules, CA, USA). Cytotoxicity was calculated using the following formula: % cytotoxicity = [(experimental - effector spontaneous - target spontaneous)/(target maximum - target spontaneous)] × 100.

Serum cytokine concentrations were measured using the Bio-Plex cytokine assay kit (Bio-Rad Laboratories, Inc., Hercules, CA, USA) according to the manufacturer’s protocol. Data analysis was performed with Bio-Plex Manager 6.1 software (Bio-Rad Laboratories, Inc.).

### Safety assessment

Adverse events, drug use, and therapy treatment were all recorded during the study. Hematology lab tests, blood chemistry assays, and urinalysis were performed with a Coulter STKS hemocytometer (Beckman Coulter Inc., Fullerton, CA, USA), a Hitachi 7150 automated analyzer (Hitachi Ltd., Tokyo, Japan), and a Clinitek Atlas automated urine chemistry analyzer (Siemens Healthcare Diagnostics, NY, USA), respectively. All analyses were performed at Korea Biomedical Laboratory. Blood pressure and pulse rate were measured with an Omron HEM-7051 device (Omron Healthcare, Kyoto, Japan); body temperature was measured with an infrared thermometer (Thermoscan IRT-4020, Braun Corporation, Kronberg, Germany).

### Randomization

An independent, study-blinded statistician prepared a computer-generated randomization scheme allowing for randomization in blocks. Sequentially numbered containers with either RBEP or placebo were obtained from the manufacturer and were randomly assigned to participants on the first visit. Identity codes were concealed in sequentially numbered opaque envelopes, managed by the study investigators, and monitored by clinical research associates (Neonutra Corporation, Seoul, Korea). All study personnel and participants remained blinded to the identity codes throughout the course of the study.

### Statistical analysis

This study aimed for a sample size of 32 in each group, in order to achieve a statistical power of 80% (*P* <0.05, two-tailed test). Considering a predicted drop-out rate of 20% during the study, 40 participants were enrolled in each group.

All analyses were performed using SAS software, version 9.2 (SAS, Inc., Cary, NC, USA). Intention-to-treat (ITT) analysis and per-protocol (PP) analysis were performed; however, only the results of PP analysis were presented since there were no differences between ITT and PP analysis. Mean changes of continuous variables from weeks 0 to 4 and from weeks 0 to 8 between the RBEP and the placebo groups were compared using the independent t-test. Nominal variables between the groups were indicated with the number of participants and percent distributions. Differences between the groups were analyzed for significance using the Chi-square test, Fisher’s exact test, and McNemar’s test. *P*-values < 0.05 were considered statistically significant.

## Results

### Participant characteristics

**Figure 1:**
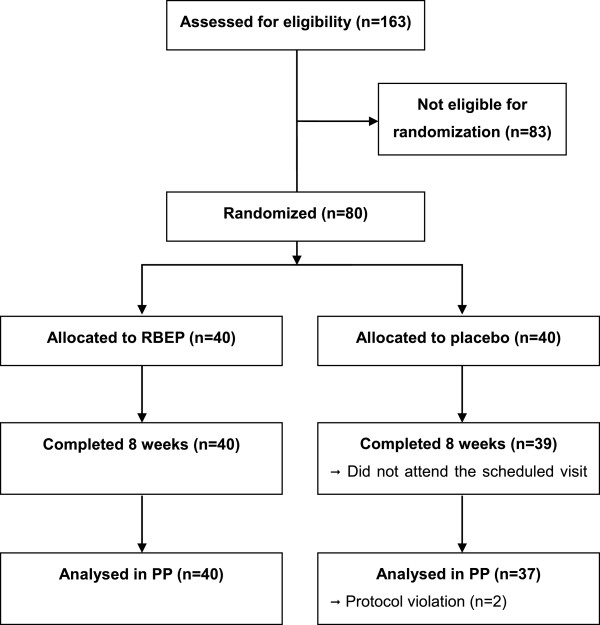
**Schematic diagram of study profile.** RBEP, rice bran fermented with *Lentinus edodes.*

A schematic diagram of the study workflow is shown in Figure 1. In the placebo group, 1 participant did not show up on the second visit, and 2 participants were removed from the study due to protocol violations. Compliance was not significantly different between the RBEP and placebo groups (89.1 ± 10.8% vs. 91.7 ± 8.2%; *P* = 0.240). During the study, no significant changes in nutrient intake between the two groups were observed (data not shown). The two groups also did not exhibit any significant differences with respect to age, WBC count, level of thyroid-stimulating hormone, sex, BMI, presence of family history related with immune disorders, number of married or unemployed participants, or number of current smokers or current drinker (Table 2). Thirteen participants in RBEP group and eleven in placebo group exercised regularly, more than 3 times per week with 30 min or more. In addition, two participants had musculoskeletal disorder, one participant had reproductive system disorder, and two participants had respiratory disorders within 6 months before the study. However, none of the disorders were severe and no one had current health problems.

**Table 2:** **Baseline characteristics of participants taking either rice bran fermented with *Lentinus edodes *** **(RBEP) or placebo**^**1**^

	**RBEP (n = 40)**	**Placebo (n = 37)**	**P value**^**2**^
Age (yrs)	32.58 ± 10.74	34.97 ± 11.90	0.356
WBC (× 10^9^/L)	5.96 ± 1.14	5.85 ± 1.40	0.709
TSH (mIU/L)	1.85 ± 1.37	1.69 ± 0.81	0.543
Height (cm)	169.05 ± 9.13	167.94 ± 9.61	0.591
Weight (kg)	65.04 ± 11.71	67.29 ± 12.76	0.402
BMI (kg/m^2^)	22.74 ± 2.86	23.54 ± 2.80	0.214
Female, n (%)	17 (42.5)	14 (37.8)	0.677
Family history, n (%)	3 (7.5)	2 (5.4)	0.709
Medical history, n (%)	2 (5.0)	2 (5.4)	0.936
Married, n (%)	11 (27.5)	10 (27.0)	0.963
Employed, n (%)	17 (42.5)	16 (43.2)	0.948
Smoking, n (%)	7 (17.5)	7 (18.9)	0.872
Drinking, n (%)	31 (77.5)	28 (75.7)	0.850
Exercise, n (%)	13 (32.5)	11(29.7)	0.793
Education			
≤ High school, n (%)	4 (10.0)	6 (16.2)	
University, n (%)	10 (25.0)	12 (32.4)	0.459
Postgraduate school, n (%)	26 (65.0)	19 (51.4)	

^1^Values shown are means ± standard deviation or numbers of participants (percentage distribution), as appropriate. WBC, white blood cell; TSH, thyroid-stimulating hormone; BMI, body mass index.

^2^P values were determined by the independent t-test, the chi-square test, or Fisher’s exact test.

### Primary and secondary outcomes

At baseline, primary, NK cell activity and secondary outcomes, levels of cytokines were not significantly different between the two groups (Table 3). RBEP supplementation significantly increased IFN-γ level compared to the placebo group at week 8 (*P* = 0.012). However, RBEP supplementation had no significant effects on the levels of IL-2, IL-4, IL-10, IL-12, or TNF-α compared with placebo treatment. RBEP supplementation enhanced NK activity within the RBEP group, but did not enhance NK cell activity when the RBEP group was compared with the placebo group.

**Table 3:** **Outcome measurements of participants taking either rice bran fermented with *Lentinus edodes *** **(RBEP) or placebo**^**1**^

	**RBEP (n = 40)**			**Placebo (n = 37)**		
	**Week 0**	**Week 4**	**Week 8**	**Week 0**	**Week 4**	**Week 8**
Natural killer cell activity (%)	11.48 ± 7.93	14.44 ± 6.66	14.67 ± 7.62	9.96 ± 6.42	12.50 ± 6.23	15.25 ± 7.97
Interferon-γ (pg/mL)	27.98 ± 15.10	28.48 ± 14.47	35.56 ± 17.66^2^	26.19 ± 11.61	26.14 ± 11.55	27.04 ± 12.51^2^
Interleukin-2 (pg/mL)	5.66 ± 7.22	5.63 ± 6.42	4.51 ± 3.99	5.97 ± 11.95	9.04 ± 28.90	4.94 ± 5.73
Interleukin-4 (pg/mL)	0.07 ± 0.06	0.08 ± 0.08	0.08 ± 0.09	0.08 ± 0.12	0.08 ± 0.13	0.08 ± 0.11
Interleukin-10 (pg/mL)	2.63 ± 9.14	3.83 ± 13.10	1.53 ± 5.14	9.30 ± 44.63	9.26 ± 44.14	8.59 ± 43.59
Interleukin-12 (pg/mL)	16.91 ± 62.78	20.55 ± 73.79	9.33 ± 24.32	11.12 ± 36.21	12.77 ± 40.62	10.62 ± 37.61
Tumor necrosis factor-α (pg/mL)	0.66 ± 0.64	0.91 ± 0.88	0.57 ± 0.53	0.57 ± 0.50	0.77 ± 0.65	0.49 ± 0.37

^1^Values shown are means ± standard deviation.

^2^*P*-values < 0.05 for differences between the RBEP and placebo groups from weeks 0 to 8 were determined using the independent t-test.

### Safety assessment

Minor adverse events were reported in 11 participants (26.19%, 15 cases) in the RBEP group and 18 participants (42.86%, 23 cases) in the placebo group; this difference was not significant (*P* = 0.108; Table 4). The numbers of adverse events judged to be possibly related to interventions, probably not related to interventions, and definitely not related to interventions were 3, 2, and 3 in the RBEP group and 1, 10, and 19 in the placebo group, respectively.

**Table 4:** **Adverse events frequently occurring in participants taking either rice bran fermented with *Lentinus edodes *** **(RBEP) or placebo**

**Adverse event**	**RBEP (n = 40)**	**Placebo (n = 37)**
	**n (%)**	**n (%)**
Constipation	2 (13.33)	1 (4.35)
Diarrhea	1 (6.67)	0 (0.00)
Dyspepsia	1 (6.67)	2 (8.70)
Gaseousness	1 (6.67)	0 (0.00)
Gingiva hypertrophia	1 (6.67)	0 (0.00)
Nausea	0 (0.00)	1 (4.35)
Stomachache	1 (6.67)	1 (4.35)
Generalized aching	1 (6.67)	4 (17.39)
Herpes labials	0 (0.00)	2 (8.70)
Fractured rib	0 (0.00)	1 (4.35)
Headache	1 (6.67)	2 (8.70)
Common cold	4 (26.67)	4 (17.39)
Enlarged tonsils	0 (0.00)	3 (13.04)
Eczema	0 (0.00)	1 (4.35)
Pruritus	2 (13.33)	0 (0.00)
Depilation	0 (0.00)	1 (4.35)

The average platelet count was significantly lower for the RBEP group than for the placebo group only at baseline, and the average WBC count was significantly decreased in the RBEP group (*P* = 0.016, Table 5). However, no significant differences in other hematological parameters, blood chemistry values, weight, blood pressure, pulse rate, or body temperature were observed between the two groups. Urinalysis also did not reveal any significant differences between the two groups (data not shown). All hematological parameters, blood chemistry values, and urinalysis results remained within their normal ranges.

**Table 5:** **Safety assessments in participants taking either rice bran fermented with *Lentinus edodes *** **(RBEP) or placebo**^**1**^

	**RBEP (n = 40)**			**Placebo (n = 37)**		
	**Week 0**	**Week 4**	**Week 8**	**Week 0**	**Week 4**	**Week 8**
Red blood cell count (× 10^12^/L)	4.33 ± 0.43	4.47 ± 0.50	4.47 ± 0.44	4.38 ± 0.43	4.58 ± 0.41	4.56 ± 0.42
White blood cell count (× 10^9^/L)	5.96 ± 1.14	5.82 ± 1.21	5.52 ± 1.12	5.85 ± 1.40	5.79 ± 1.16	5.85 ± 1.20
Hemoglobin (g/L)	140.10 ± 13.10	138.70 ± 14.00	139.60 ± 13.30	140.10 ± 12.60	140.50 ± 12.00	140.30 ± 12.70
Hematocrit (%)	41.45 ± 4.13	42.38 ± 4.48	42.09 ± 3.85	41.46 ± 3.90	42.76 ± 3.62	42.34 ± 3.99
Platelet count (× 10^9^/L)	236.13 ± 39.60^2^	242.18 ± 46.44	243.35 ± 45.91	259.11 ± 61.15^2^	266.19 ± 55.50	267.97 ± 46.08
Segmented neutrophils (%)	54.75 ± 7.61	53.25 ± 5.79	53.30 ± 7.13	54.65 ± 8.47	54.91 ± 5.77	54.97 ± 5.95
Lymphocytes (%)	34.83 ± 7.29	37.60 ± 5.95	36.80 ± 6.65	36.27 ± 7.25	35.78 ± 5.80	35.05 ± 5.52
Monocytes (%)	6.63 ± 1.51	5.90 ± 1.22	6.53 ± 1.45	6.19 ± 1.29	5.84 ± 1.48	6.30 ± 1.61
Eosinophils (%)	2.78 ± 1.21	2.38 ± 0.90	2.38 ± 1.17	2.51 ± 1.26	2.32 ± 1.08	2.73 ± 1.35
Basophils (%)	1.03 ± 0.48	0.88 ± 0.52	1.00 ± 0.55	0.84 ± 0.55	0.97 ± 0.44	0.95 ± 0.57
AST (U/I)	21.25 ± 6.41	21.88 ± 7.77	23.58 ± 9.67	25.24 ± 11.25	24.16 ± 7.30	25.65 ± 10.63
ALT (U/I)	17.20 ± 14.66	17.50 ± 22.40	25.53 ± 27.40	22.51 ± 16.31	21.24 ± 13.41	27.57 ± 19.08
Total protein (g/L)	72.20 ± 4.00	72.60 ± 5.00	73.50 ± 3.90	73.20 ± 3.80	72.50 ± 5.30	74.20 ± 3.70
Glucose (mmol/L)	4.43 ± 0.37	4.31 ± 0.30	4.86 ± 0.45	4.55 ± 0.37	4.44 ± 0.31	4.97 ± 0.38
Total cholesterol (mmol/L)	4.74 ± 0.81	4.64 ± 0.81	5.00 ± 0.84	4.87 ± 0.90	4.81 ± 0.90	5.22 ± 0.88
Blood urea nitrogen (mmol/L)	4.33 ± 0.81	4.51 ± 1.16	4.84 ± 1.12	4.45 ± 1.07	4.59 ± 1.21	5.03 ± 1.10
Creatinine (μmol/L)	60.25 ± 10.68	68.63 ± 15.25	61.01 ± 10.68	61.77 ± 14.49	67.11 ± 14.21	62.53 ± 14.00
Uric acid (μmol/L)	294.43 ± 63.64^2^	281.34 ± 55.91	311.08 ± 73.16	318.81 ± 74.35	293.24 ± 60.07	342.60 ± 77.32
Calcium (mmol/L)	2.24 ± 0.08	2.27 ± 0.06	2.25 ± 0.06	2.23 ± 0.08	2.26 ± 0.07	2.24 ± 0.06
Phosphorus (mmol/L)	1.14 ± 0.14	1.24 ± 0.10	1.22 ± 0.09	1.15 ± 0.12	1.21 ± 0.12	1.22 ± 0.11
SBP (mmHg)	116.43 ± 15.20	116.03 ± 11.76	120.15 ± 13.55	118.87 ± 11.73	117.46 ± 12.05	121.32 ± 13.54
DBP (mmHg)	71.73 ± 9.73	71.98 ± 9.74	74.85 ± 9.73	71.11 ± 8.97	73.65 ± 9.39	75.59 ± 10.39
Pulse rate (beats/min)	76.48 ± 9.93	75.53 ± 10.89	75.50 ± 10.61	73.22 ± 11.01	75.54 ± 10.81	74.68 ± 10.24
Body temperature (°C)	36.24 ± 0.38	35.95 ± 0.35	35.97 ± 0.44	36.29 ± 0.41	36.15 ± 0.44	36.04 ± 0.42

^1^Values shown represent means ± standard deviation. AST, Aspartate aminotransferase; AT, Alanine aminotransferase; SBP, Systolic blood pressure; DBP, Diastolic blood pressure.

^2^*P*-values < 0.05 for differences between the RBEP and placebo groups at week 0 were determined using the independent t-test.

## Discussion

This was the first randomized, double-blind, placebo-controlled, parallel-group study investigating the immune-modulatory effects of RBEP in healthy adults. This group study revealed that RBEP supplementation significantly increased IFN-γ levels compared with a placebo group, but RBEP supplementation did not appear to enhance NK cell activity as compared to placebo group. Importantly, RBEP supplementation did not cause any significant adverse effects in the present study.

In contrast to these results, previous studies have reported that an enzymatically modified version of rice bran increases NK cell activity; this finding is potentially important because NK cells play important roles in the immune system, including defending against cancer [2,9,10,13,15]. Furthermore, RBEP supplementation in mice has been shown to exhibit anticancer effects by enhancing NK cell activity [2]. Arabinoxylan, mainly xylose polymer from rice bran, have also been reported to increase NK cell activity in patients with various cancers [9,13] and in mice bearing Ehrlich ascites [10] and renal carcinoma [16]. The most likely reason for these discrepancies is that the present study examined healthy subjects, whereas the previous studies all examined the effects of RBEP in the context of cancer. McDermott et al. [17] concluded that arabinoxylan supplementation was not effective for treating chronic fatigue syndrome, an assessment thought to indirectly measure NK cell activity. Cancer patients have been suggested to have reduced NK cell activity compared with healthy individuals [18,19]; thus, significant effects on NK cell activity in healthy individuals may be difficult to observe. In the present study, NK cell activity was significantly increased within the RBEP group after supplementation compared with the same group before supplementation; however, this effect was no longer significant when the RBEP group was compared with the placebo group. Only one previous study in healthy adults reported that supplementation of an arabinoxylan obtained from rice bran increased NK cell activity, although this study did not include a placebo control [11].

In agreement with the results obtained in the present study, previous studies have reported that an arabinoxylan isolated from rice bran significantly increased IFN-γ levels in patients with multiple myeloma [9], in mice bearing Ehrlich ascites [10], and in *in vitro* studies [11,12,20]. Furthermore, polysaccharides from *Angelica sinensis* and from the root of *Sanguisorba officinalis* have been shown to increase IFN-γ production in spleen cells [21] and tumor-bearing mice [22], respectively. This immune-enhancing action of arabinoxylan has been proposed to be related to the increased IFN-γ secretion accompanying NK cell activation [11]. However, IFN-γ is produced not only by NK cells, but also by T and B cells [23].

Previous studies have also reported that RBEP supplementation activates macrophages, both *in vivo* and *in vitro*[5,8]*.* An arabinoxylan obtained from rice bran, the green leaves of *Litsea glutinosa*, and wheat bran have been shown to increase macrophage activity, either *in vitro*[24,25] or in tumor-bearing mice [26]. In addition, an arabinoxylan isolated from rice bran increased CD4+ T cell function in human monocyte-derived dendritic cells [20], and increased T and B cell proliferation in both humans with and without cancer [13,27]. Previous studies have consistently reported that polysaccharides from evening primrose, *Curcuma xanthorrhiza,* and *Angelica sinensis* increase macrophage activity and/or T cell proliferation, either *in vivo* or *in vitro*[21,28,29]. These data, in combination with the data presented in this study, suggest that the increased production of IFN-γ resulting from RBEP supplementation may be mediated by either macrophages, B cells, or T cells, and it is probably not mediated by NK cells.

Kim et al. showed that RBEP supplementation did not exhibit hematopoietic toxicity in a mouse model of solid tumors, thereby providing important evidence that RBEP is safe and could potentially be used to both prevent and treat cancer [2]. Increased WBC counts have been observed in patients with inflammation [30], and have been shown to be associated with high cancer mortality [31]. In the present study, REBP supplementation led to a negligible decrease in WBC count; however, participant WBC counts remained within the normal range, and thus this finding is clinically non-significant.

## Conclusions

This was the first randomized, double-blind, placebo-controlled, parallel-group study investigating the immune-modulatory effects of RBEP in healthy adults. The data presented here suggest that RBEP supplementation significantly increases IFN-γ production, but does not enhance NK cell activity; furthermore, RBEP supplementation is not associated with any significant adverse effects.

## Abbreviations

ALT: Alanine aminotransferase; AST: Aspartate aminotransferase; BRM: Biological response modifier; IFN: Interferon; IL: Interleukin; ITT: Intention-to-treat; NK: Natural killer; PBMC: Peripheral blood mononuclear cell; PP: Per-protocol; RBEP: Rice bran fermented with *Lentinus edodes*; TNF: Tumor necrosis factor; TSH: Thyroid-stimulating hormone; WBC: White blood cell.

## Competing interests

The authors declare that there are no conflicts of interests.

## Authors’ contributions

JYC conducted the research and wrote the manuscript; JYC and DYK analyzed the data and took responsibility for the integrity of the data and the accuracy of the data analysis; YP designed the research and had primary responsibility for the final content. All authors read and approved the final manuscript.
